# Prevalence of and Factors Associated with Sports Injuries in 11,000 Japanese Collegiate Athletes

**DOI:** 10.3390/sports12010010

**Published:** 2023-12-28

**Authors:** Takeshi Kimura, Aleksandra Katarzyna Mącznik, Akira Kinoda, Yuichi Yamada, Yuki Muramoto, Yoshinori Katsumata, Kazuki Sato

**Affiliations:** Institute for Integrated Sports Medicine, Keio University School of Medicine, Tokyo 160-8582, Japan; takeshikimura4@gmail.com (T.K.); alexmacznik@gmail.com (A.K.M.); akirakiak@keio.jp (A.K.); yy.onlyone@gmail.com (Y.Y.); yukimuramoto1019@gmail.com (Y.M.); goodcentury21@gmail.com (Y.K.)

**Keywords:** sports injuries, university students, surveillance, period prevalence, athletic injuries, college athletes, cross-sectional survey

## Abstract

Background: To establish the 1-year prevalence of sports injuries and explore associations of various factors with a sports injury in Japanese collegiate athletes. Methods: The data were collected through a web-based survey of Japanese collegiate athletes associated with UNIVAS (Japan Association for University Athletics and Sport). The survey questions asked about athletes’ personal characteristics, sports participation, and injuries sustained within the previous year. Follow-up questions on the details regarding the three most serious injuries were asked. Differences in proportions of athlete characteristics between males and females and between injured and uninjured were explored with the chi-square test. Factors associated with sustaining an injury were determined with regression analysis. Results: The prevalence of injuries among Japanese collegiate athletes is high, and most of the sustained injuries require athletes to take a considerable time off training and competition indicating their severity. Athletes from year two and higher at the university, overweight or obese, training more often per week, and with longer sports experience were more likely to sustain an injury within the previous year. Conclusions: There is compelling evidence to suggest that excessive training and insufficient recovery may be contributing to their increased risk of injury. These findings underscore the importance of implementing evidence-based training programs and recovery strategies to mitigate injury risk and optimize performance outcomes among this population.

## 1. Introduction

Sports injuries have a profound impact on both individuals and society as a whole. Athletes who suffer injuries often experience negative effects on their performance, physical and mental health, and their ability to participate in sports long-term [1]. These injuries can also increase the likelihood of reinjury, and may cause mental health issues such as blame, impatience, fear of performance, and pressure to return to training [2]. In addition, serious sports and recreational injuries can result in a significant decrease in physical activity levels, which can lead to negative health outcomes in the future [3]. Furthermore, sports injuries have a substantial economic burden due to both direct and indirect costs. This burden affects not only top-level athletes but also recreational athletes of all ages, as their injuries may impact their health and ability to work [4]. Overall, sports injuries have a significant impact on athletes’ physical, psychological, and financial well-being, making preventive measures necessary to reduce the risks leading to injuries, particularly those that are serious and long-term [3,4].

To minimize the risk of injuries, various strategies can be employed, such as altering rules, mandating protective equipment, educating athletes and coaches, adjusting training regimens, and developing injury prevention programs [5,6]. Implementation of these strategies may be necessary on a global or international scale, while others may be the responsibility of individual athletes. This complexity adds to the challenge of addressing the issue comprehensively.

Sports injury prevention strategies at a country level have been introduced internationally on various levels of sports and with varying success [6]. In Japan, sports injuries have been monitored in school settings (elementary school, junior high school, high school, etc.) by the Japan Sports Council [7]. Sport-specific organizations have also made efforts in sports injury risk reduction within their disciplines [8,9]. In 2019, The Japan Association for University Athletics and Sport (UNIVAS) was established with a total of about 140,000 collegiate athletes associated. This allowed a national-level effort to investigate and subsequently reduce injuries in the collegiate population with this investigation as the first step.

Understanding what the common injuries and associated factors is the first step on the path to reducing the risk of sports injury in the future [10,11]. Therefore, the intent of this study was to collect data on the 1-year prevalence of sports injuries and explore associations of various factors with a sports injury in Japanese collegiate athletes.

## 2. Materials and Methods

In this cross-sectional observational study, the data were collected through the web-based survey available from June 2022 to August 2023. The study was approved by the Ethics Committee of Keio University (approval number: 20211158). All participants provided online informed consent before participating. The study was conducted in accordance with the principles of the Declaration of Helsinki [12] and reported according to the STROBE-SIIS consensus statement [13]. The sample size of 372 participants was determined using the formula devised by Charan and Biswas [14], with the expected proportion in the population drawn from a study on Japanese collegiate handball players [9].

Potential participants were invited from 219 universities and 36 sports organizations associated with UNIVAS. The universities’ sports departments and sports organizations were invited through letters and phone calls. After the initial interest was shown, the sports managers or athletic trainers were asked to distribute the link to the study’s information sheet and consent form to the athletes. After the athlete read the information sheet and agreed to take part in the study, they were invited to take the survey.

The survey was placed on the purposefully built website (https://enquete.cc/q/BC2XC8A8; accessed on 1 June 2022). The questions were adopted from the recently published Japanese Society of Clinical Sports Medicine and Japanese Society for Athletic Training consensus document [15] and modified to best suit the collegiate population. 

The questions asked about athlete characteristics (age, year of study, weight, height, dexterity), sports participation (sport played, sporting experience, hours of training per week, competitions per season), and injuries sustained within the last year (April 2021–March 2022). Follow-up questions about the details regarding the three most serious injuries were asked (location, severity, type, part of the season when injured, onset in match or training, mechanism, and time lost from sports participation). 

Injury severity was defined based on time lost from training/competition and classified using approach as follows: minimal—0 days missed; mild—1 day—1 week lost; moderate—1 week—1 month lost; severe—1 month—6 months, very severe—more than 6 months lost [13,16].

Type of an injury was defined as new—a new injury occurred, recurrent—the injury disappeared before but occurred again, exacerbated—symptoms worsened [15]. 

Mechanism of an injury was defined as direct—the force to the injured area was applied directly by another athlete or object, indirect—the force was applied indirectly by another athlete or object, or non-contact—when the injury occurred without direct or indirect contact with another athlete or object [15]. Each injury was attributed to a singular primary mechanism.

All statistical analyses were performed with SPSS (IBM Corp. Released 2021. IBM SPSS Statistics for Macintosh, Version 28.0. IBM Corp, Armonk, NY, USA). Continuous data were summarized with mean and standard deviation. For categorical variables, frequencies and percentages were calculated. A *p*-value of <0.05 was considered statistically significant.

Period prevalence (1 year) was calculated by dividing the number of athletes injured by the number of all athletes and multiplied by 100%. We compared the differences in proportions of injury characteristics (e.g., location, severity, type, onset, mechanism, time lost) between females and males using the chi-square test (Pearson’s chi-square, Fisher’s exact test, or Fisher’s exact test with Monte Carlo estimates, as appropriate) for categorical data. We assessed the associated factors (participant demographics, sports participation) with injury occurrence using regression models. For each of the outcome variables, we estimated odds ratios (ORs) with 95% CIs using a logistic regression approach.

## 3. Results

### 3.1. Participants Characteristics

**Figure 1:**
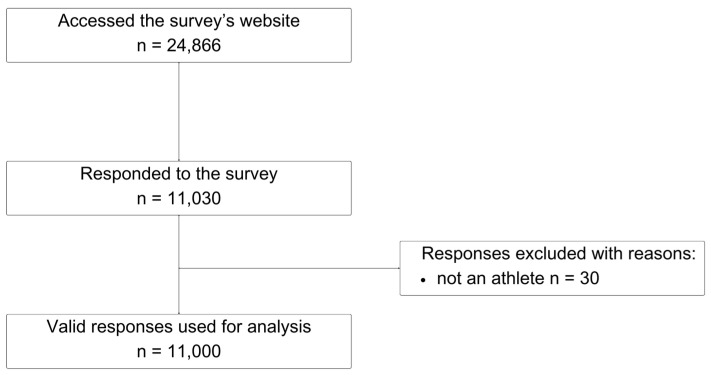
Flow diagram of the study.

Out of 24,866 unique clicks to the survey’s webpage, a total of 11,030 responses were recorded. However, 30 of these were submitted by non-athletes and therefore excluded, leaving 11,000 valid responses (response rate of 44.2%). A flow diagram of the study is shown in Figure 1. 

The summary of the survey participants’ characteristics is in Table 1. The athletes who responded to this survey were on average 19.9 (±1.3) years of age and had about 8 years of experience in sport. They played mainly team sports, practiced 5.2 days per week, and mostly played up to 10 matches per season. The athletes surveyed in this study represented 84 sports, with the most represented being lacrosse, softball, and baseball. Seventy-four sports were represented by less than 2% athletes each (e.g., tennis n = 230, handball n = 212, rowing n = 195). Half of the athletes (n = 5500) surveyed in this study, experienced at least one injury in the previous year (April 2021–March 2022) with 45% reporting multiple injuries within the previous year. 

### 3.2. Injuries’ Characteristics

The 5500 athletes in this study reported 8211 injuries, mostly (65%) moderate, severe, or very severe in nature. Characteristics of the reported injuries are detailed in Table 2. 

Females experienced significantly higher proportions of ankle, knee, and lower leg injuries than males, and a higher proportion of their injuries were minimal or mild in severity than in males (43% vs. 31%). Males experienced a significantly higher proportion of head, neck, shoulder, elbow, and hand injuries than females, and a higher proportion of their injuries were moderate, severe, or very severe in nature when compared to females (69% vs 57%).

Overall, most injuries occurred in training, in season, and resulted in some time lost from training or competition. One-third of injuries (31.45%) were recurrent or exacerbated, and not new.

Males reported a significantly higher proportion of new injuries, occurring in season, because of direct contact with another athlete or object, and resulting in time lost than females who reported a significantly higher proportion of exacerbated injuries, sustained pre- or post-season, due to a non-contact mechanism, with a higher proportion of injuries not requiring time off training or competition.

### 3.3. Injury Prevalence and Associated Factors

An overall 1-year sports injury prevalence reported by Japanese collegiate athletes in this study was 50.01% (5500 injured out of 10,998 athletes). Sports injury prevalence in males (52.02%) was significantly higher than in females (46.65%). Differences in the proportion of characteristics between injured and uninjured athletes are presented in Table 3.

A higher proportion of males reported injuries than females. A significantly higher proportion of first-year athletes stayed injury-free but a significantly higher proportion of year 2 athletes or above reported sustaining an injury. A higher proportion of uninjured athletes was of normal weight or underweight, whereas injured students reported a higher proportion of overweight and obese BMIs. A higher proportion of uninjured athletes trained 1–4 days while a higher proportion of injured athletes reported training 5–7 days per week. Proportions between injured and uninjured athletes in matches/competitions differed but the pattern was unclear.

### 3.4. Factors Associated with Sustaining an Injury

Factors associated with sustaining injury within the previous year are presented in Table 4. 

The sex of the athlete, age, and the number of matches/competitions in a season were not related to sustaining an injury but year at university, BMI, years of sports experience, and a number of practice days per week were. Athletes were 1.5 and 1.7 times more likely to get injured if they were overweight or obese, respectively. Being a second-year student or older increased the risk of sustaining injury by 1.7–1.9 times; similarly, every year of sports experience increased the risk by 1.021. Athletes training 5 days per week or more were 2.5 times more likely to sustain an injury than students that trained 4 days per week or less.

## 4. Discussion

Half of Japanese collegiate athletes surveyed in this study sustained at least one injury during the previous year with the majority of these injuries being moderate or severe in nature and requiring considerable time off training and competition. Athletes from the second university year or higher, overweight or obese, who trained more often per week, and with longer sports experience were more likely to get injured within the previous year.

### 4.1. Prevalence of Sports Injuries

Prevalence of injuries among Japanese collegiate athletes was high with half of the athletes reporting an injury, and with 45% of athletes sustaining multiple injuries. The prevalence of sports injuries in Japanese collegiate athletes exceeds numbers reported for general and sports populations in other countries. In Germany, 3.1% of adults reported sustaining a sports injury during the previous year, and this corresponded to an annual injury rate of 5.6% among those engaging in regular recreational physical activity [17]. In Denmark, 18.4% of adults and 19.3% of children reported having had a sports injury within the past 12 months, which was considered ‘very frequent’ [18]. In a small study of Finnish adolescents, 60.3% of sports club members and 30.8% of non-members reported at least one acute or overuse injury in the previous year [19]. Brazilian athletes from one university reported an injury prevalence of 49% over the 6–10 years of their medical studies [20], not within a year like the athletes in the current study. In a one-year prospective study of one geographical region in Spain, 40.4% of adolescent athletes from different sports sustained injury within the previous year [21]. 

The high number of sports injuries in the Japanese collegiate population found in this study reveals a concerning trend of increasing injuries as the athletes move through the educational system. In Japan, athletes are mostly developed in schools’ clubs and ‘circles’. A previous study has found that 54% of surveyed Japanese collegiate athletes reported experiencing acute or overuse sports injury in their lifetime [22]. Moreover, the proportion of injured athletes consistently increased with age through lower elementary school (4%), upper elementary school (21%), junior high school (35%), and high school (41%) [22]. Addressing the sports injury problem in Japan needs to account for the pattern of increasing injuries as athletes progress from primary and junior high to high school and university sports.

Injury patterns in Japanese collegiate athletes differ between male and female athletes. Previous literature reported on differences in sports injuries between males and females [23] calling for accounting for sex differences in preventive measures. An interesting finding of this study was that upper limb, neck, and head injuries were more prevalent in male athletes, while females reported more lower limb injuries. Further sex differences in proportions of injuries were found in mechanism and part of the season the injuries were sustained. These findings are clear indications that sex differences exist but how to address these differences will depend on a particular sport. 

### 4.2. Time Lost and Severity of Injuries

The findings of this study demonstrated an extremely high proportion (82.5%) of injuries causing time lost from training and/or competition among Japanese collegiate athletes. This proportion is considerably higher than that reported in previous studies, for example, only 49.0% of ball-contact injuries [24] in NCAA ball athletes, 48% in NCAA collegiate athletes from 25 sports [25], and only 49.8% of combat sports injuries sustained during the Olympic tournament led to time lost [26].

Moreover, Japanese collegiate athletes reported a very high proportion of severe (26.2%)—1–6 months of time lost—and very severe (6.3%)—>6 months lost—injuries. For comparison, only 6.6% of ball-contact injuries required more than 3 weeks of sports cessation from collegiate ball sports [24], and 12.9% of combat Olympic tournament injuries required more than 28 days of sports cessation. In professional cyclists, only 5.3% of injuries required more than 28 days of time lost [27]. Only 9.5% of all injuries in total were reported as severe in NCAA collegiate athletes from 25 sports [28]. The examples clearly show that Japanese collegiate athletes may carry a higher burden of severe injuries than their counterparts in other countries.

The differences in data collection methods (the current study being a survey of volunteering athletes versus other studies using medical/insurance records) can potentially explain some of the discrepancies, as severely injured athletes may have been more likely to participate in the survey than uninjured or less severely injured athletes (selection bias). 

Males had proportionally more moderate and severe injuries than females in this study, but females reported more minimal and mild injuries. No difference between sexes was found in the proportion of very severe injuries. This finding is unique as Lystad et al. found no difference between sexes in combat sports athletes injured while competing at the Olympics, and Fraser et al. found no difference in severe ball-contact injuries between males and females [24,26]. One possible explanation for the differences in the severity of injuries between male and female Japanese collegiate athletes may be related to differences in training culture. While male and female combat sports athletes often train together, using the same methodology, there are cultural differences in training among Japanese female and male athletes. Lower participation rates of females in strength training, strength training being perceived as not suitable for the female body, and other sex-biased beliefs in coaches may lead to differences in physical preparation, possibly resulting in differing injury patterns [29]. 

Severe injuries may be a reason to end an athletic career and can have serious health and mental long-term consequences [30,31]. The results of this study suggest that injury prevention strategies should be developed and implemented among Japanese collegiate athletes to reduce the frequency of severe and very severe injuries. These strategies should take into account differences in training culture and focus on improving physical preparation and injury prevention techniques, particularly among male athletes.

### 4.3. Factors Associated with Sports Injuries

The current study found that longer experience in sports, higher year at university, higher frequency of training per week, and status of overweight or obese BMI, yielded higher odds of sustaining an injury within the previous year in Japanese collegiate athletes. 

The first three factors seem to be indicating overtraining or under-recovery as possible underlying reasons for the increased risk of injury in the studied population. Years of experience in sports and year at university should be considered together because as the athletes will go through the Japanese education system, they simultaneously gain experience in sports. The frequency of training per week has been reported to steadily increase from early elementary to high school, as was the prevalence of sports injuries [22]. Additionally, a high number of injuries in the cohort of collegiate athletes can be related to a strong culture of dedication to one sport in Japanese athletes where only 10–17% of athletes in junior high school and 1–9% in a high school play/sample other sports [22,32]. Playing multiple sports in junior high school was associated with fewer overuse injuries in junior high school and high school [31]. Early specialization was shown to have a detrimental effect on injury rates in several sports internationally before and seems to be a valid factor influencing the health and performance of collegiate and senior athletes [33,34]. Moreover, one-third of all injuries were reported in this study as recurrent or exacerbated and therefore not new. The number of lifetime overuse injuries was reported at 68% in another sample of Japanese collegiate athletes [21]. These, further support the long-term overtraining as a factor associated with high injury prevalence reported in this study. 

In this study, overweight or obese BMI was associated with a higher risk of sustaining sports injury among Japanese collegiate athletes. Postulated mechanisms of the influence of increased body mass include impaired balance and postural control [35], and increased severity of exercise-induced muscle damage that may lead to increased levels of circulating inflammatory molecules and cause chronic low-grade inflammation [36]. Therefore, careful management of training load and recovery as well as additional balance training should be considered in overweight and obese athletes.

### 4.4. Limitations

This study has several limitations that must be considered when interpreting the results. First, the retrospective design of this study opens the possibility for recall bias where athletes may not accurately remember all injuries they experienced or may over- or underestimate the severity of injuries. To mitigate this limitation, we only asked about the three most serious injuries, which may have reduced recall bias to some extent. Furthermore, the retrospective design may limit the ability to establish causal relationships between injuries and other variables of interest. Another limitation is the relatively low response rate, as only 11,000 athletes out of a total of 140,000 UNIVAS athletes responded to the survey. This may have introduced selection bias, which may limit the generalizability of the findings to other populations. However, this number well exceeded the calculated sample size.

Despite these limitations, the present study provides valuable insights into injury patterns among Japanese athletes and highlights the need for injury prevention programs in this population. Future research should aim to address these limitations and provide a more comprehensive understanding of injury patterns and risk factors among athletes in prospective and ongoing designs. 

## 5. Conclusions

Prevalence of injuries among Japanese collegiate athletes is high, and most of the sustained injuries require athletes to take considerable time off training/competition indicating their severity. The number of years of experience in sports, year at the university, frequency of training per week, and BMI were associated with sustaining an injury within the previous year in Japanese collegiate athletes. 

Based on the analysis of factors associated with injuries in the previous year among Japanese collegiate athletes in this study, there is compelling evidence to suggest that prolonged excessive training accompanied by insufficient recovery may be contributing to their increased risk of injury. These findings underscore the importance of implementing evidence-based training programs and recovery strategies to mitigate injury risk and optimize performance outcomes among this population. Further research is warranted to better understand the underlying mechanisms driving this association and to develop targeted interventions to address this issue.

## Figures and Tables

**Table 1:** Characteristics of participants.

Characteristic	All	Males	Females	Sex Unspecified
	n = 11,000	n = 6848	n = 4096	n = 56
	100%	62.3%	37.2%	0.5%
Age, years; mean ± SD	19.9 ± 1.3	19.9 ± 1.4	19.8 ± 1.3	20.1 ± 1.4
Year at university; n (%)				
Year 1	3626 (33.0)	2268 (33.1)	1343 (32.8)	15 (26.8)
Year 2	2794 (25.4)	1765 (25.8)	1018 (24.8)	12 (21.4)
Year 3	2348 (21.3)	1496 (21.8)	838 (20.4)	15 (26.8)
Year 4	2145 (19.5)	1250 (18.3)	881 (21.5)	14 (25.0)
Year 5	51 (0.5)	44 (0.6)	7 (0.2)	0 (0)
Year 6	34 (0.3)	25 (0.4)	9 (0.2)	0 (0)
Height, cm; mean ± SD	168.3 ± 8.6	173.1 ± 6	160.2 ± 5.8	165.7 ± 10
Weight, kg; mean ± SD	66.2 ± 14.1	72.7 ± 13.3	55.5 ± 7.1	61.4 ± 13.7
BMI; mean ± SD	23.2 ± 3.6	21.6 ± 2.3	21.6 ± 2.3	22.3 ± 3.9
Sporting experience, years; mean ± SD	8 ± 4.9	8.1 ± 5	7.9 ± 4.8	7.7 ± 4.5
Sports; n (%)				
Lacrosse	1689 (15.4)	701 (10.2)	978 (23.9)	10 (17.9)
Softball	1243 (11.3)	300 (4.4)	994 (22.8)	9 (16.1)
Baseball	1212 (11.0)	1208 (17.6)	1 (0.0)	3 (5.4)
American Football	896 (8.1)	887 (13.0)	5 (0.1)	4 (7.1)
Soccer	884 (8.0)	697 (10.2)	184 (4.5)	3 (5.4)
Rugby	829 (7.5)	794 (11.6)	34 (0.8)	1 (0.0)
Judo	564 (5.1)	376 (5.5)	183 (4.5)	5 (8.9)
Track	531 (4.8)	386 (5.6)	143 (3.5)	2 (3.6)
Basketball	433 (3.9)	117 (1.1)	314 (7.7)	2 (3.6)
Volleyball	334 (3.0)	105 (1.5)	225 (5.5)	4 (7.1)
Other (74 sports)	2385 (21.7)	1277 (18.6)	1095 (26.7)	13 (23.2)
Practice days per week; mean ± SD	5.2 ± 1.3	5.2 ± 1.3	5.1 ± 1.3	4.8 ± 1.5
Matches/competitions per season; n(%)				
1–5 matches	6494 (58.1)	3711 (54.2)	2648 (64.6)	37 (66.1)
6–10 matches	2462 (22.4)	1673 (24.4)	781 (19.1)	8 (14.3)
11–15 matches	872 (7.9)	600 (8.8)	270 (2.5)	2 (3.6)
16–20 matches	509 (4.6)	363 (5.3)	143 (3.5)	3 (5.4)
21–25 matches	256 (2.3)	171 (2.5)	83 (2.0)	2 (3.6)
26–30 matches	153 (1.4)	98 (1.4)	54 (1.3)	1 (1.8)
>30 matches	352 (3.2)	232 (3.4)	117 (2.9)	3 (5.4)
Hand dominance; n (%)				
right	9786 (89.0)	6038 (88.2)	3704 (90.4)	46 (82.1)
left	968 (8.8)	670 (9.8)	296 (7.2)	2 (3.6)
both	210 (1.9)	119 (1.7)	85 (2.1)	6 (10.7)
Leg dominance; n (%)				
right	9256 (84.2)	5879 (85.8)	3340 (81.5)	39 (69.6)
left	1422 (12.9)	809 (11.8)	605 (14.8)	8 (14.3)
both	266 (2.4)	137 (2.0)	123 (3.0)	6 (10.7)

*p*-value of <0.05 was considered statistically significant.

**Table 2:** Characteristics of sports injuries.

Characteristic	All	Males	Females	Sex Unspecified
	n = 11,000	n = 6848	n = 4096	n = 56
	100%	62.3%	37.2%	0.5%
Reported injuries within a year; n (%)	5500 (50.0)	3562 (52.0)	1910 (46.7)	28 (50.0)
one	3280 (54.8)	2128 (31.1)	1138 (27.8)	14 (50.0)
two	1458 (13.3)	919 (13.4)	532 (13.0)	7 (12.5)
three	417 (3.7)	276 (4.0)	138 (3.3)	3 (5.4)
more	345 (3.1)	239 (3.5)	102 (2.5)	4 (7.1)
none	5500 (50.0)	3286 (48.0)	2186 (53.4)	28 (50.0)
Injury location; n	8190	5317	2825	48
	Missing 214	Missing 3	Missing 18	Missing 0
Head				
Head	246	204 *	40	2
Face	153	89	62	2
Trunk				
Lumbo-sacral, spine, buttocks	763	492	267	4
Chest	80	58	22	0
Neck	73	57 *	15	1
Upper back	46	28	18	0
Abdomen	29	18	11	0
Upper limb				
Shoulder	749	561 *	187	1
Elbow	342	258 *	83	1
Hand	249	181 *	66	2
Finger	206	133	73	0
Wrist	168	102	64	2
Thumb	103	69	33	1
Arm	83	57	26	0
Lower limb				
Ankle	1423	883	525 *	15
Knee	1167	682	476 *	9
Thigh	983	651	329	3
Toe	747	453	289	5
Lower Leg	386	221	165 *	0
Hip joint	99	57	42	0
Achilles	92	60	32	0
Severity; n	8211	5320	2843	48
Minimal (0 days lost)	1215	630	577 *	8
Mild (1D–1W lost)	1680	1014	656 *	10
Moderate (1W–1M lost)	2650	1816 *	821	13
Severe (1M–6M lost)	2147	1535 *	599	13
Very severe (>6 months lost)	519	325	190	4
Type; n	8211	5320	2843	48
new	5629	3721 *	1872	36
recurrent	2266	1439	818	9
exacerbated	316	160	153 *	3
Onset (part of the season); n				
pre-season	3372	2136	1221 *	15
in season	4100	2791 *	1288	21
post-season	580	325	249 *	6
other	159	68	85	6
Onset (match or training); n				
in match	2283	1495	782	6
in training	5928	3825	2061	42
Mechanism; n				
direct	3405	2458 *	926	23
indirect	929	620	304	5
non-contact	3875	2242	1613 *	20
Time lost; n				
yes	6771	4521 *	2209	41
no	1440	799	634 *	7

* experienced higher proportion of injuries than the other sex; a *p*-value of <0.05 was considered statistically significant.

**Table 3:** The difference in characteristics’ proportion between injured and uninjured athletes (n = 10,944).

	Injured	Uninjured		
	n = 5472 *	n = 5472 *	Pearson’s χ^2^	*p* value
Sex; n (%)	50%	50%		
Male	**3562 (65.1)**	3286 (60.1)	29.5	<0.001
Female	1910 (34.9)	**2186 (39.9)**		
Year at university; n (%)				
Year 1	1421 (26.0)	**2190 (40.0)**	257.8	<0.001
Year 2	**1480 (27.0)**	1303 (23.8)		
Year 3	**1338 (24.5)**	996 (18.2)		
Year 4+	**1233 (22.5)**	983 (18.0)		
BMI; n (%)				
Underweight (<18.5)	160 (2.9)	**240 (4.4)**	179.2	<0.001
Normal (18.5–24.9)	3750 (68.5)	**4245 (77.6)**		
Overweight (25.0–29.9)	**1175 (21.5)**	773 (14.1)		
Obese (>30.0)	**387 (7.1)**	214 (3.9)		
Practice days per week; n (%)				
1	36 (0.7)	**217 (4.0)**	578.4	<0.001
2	61 (1.1)	**242 (4.4)**		
3	229 (4.2)	**605 (11.1)**		
4	460 (8.4)	**709 (13.0)**		
5	**1314 (24.0)**	1095 (20.0)		
6	**3129 (57.2)**	2420 (44.2)		
7	**243 (4.4)**	184 (3.4)		
Matches/competitions per season; n (%)				
1–5 matches	3003 (54.9)	**3355 (61.3)**	54.9	<0.001
6–10 matches	**1347 (24.6)**	1108 (20.2)		
11–15 matches	393 (7.2)	**477 (8.7)**		
16–20 matches	**270 (4.9)**	236 (4.3)		
21–25 matches	127 (2.3)	127 (2.3)		
26–30 matches	73 (1.3)	79 (1.4)		
>30 matches	175 (3.2)	174 (3.2)		

* data for females and males only; **bold**—experienced higher proportion than the other; a *p*-value of <0.05 was considered statistically significant.

**Table 4:** Factors related to sustaining an injury (logistic regression analysis).

Factor	Odds Ratio	95%CI	*p* Value
Age	1.002	0.943–1.066	0.943
Sex			
Male	reference		
Female	0.942	0.863–1.029	0.184
Year at university			
Year 1	reference		
Year 2	**1.741**	1.538–1.970	<0.001
Year 3	**1.951**	1.651–2.306	<0.001
Year 4+	**1.760**	1.411–2.194	<0.001
BMI			
Normal (18.5–24.9)	reference		
Underweight (<18.5)	0.959	0.770–1.194	
Overweight (25.0–29.9)	**1.465**	1.313–1.635	<0.001
Obese (>30.0)	**1.700**	1.415–2.044	<0.001
Sports Experience	**1.021**	1.012–1.030	<0.001
Practice days per week			
1–4	reference		
5–7	**2.482**	2.234–2.757	<0.001
Matches/competitions per season;			
1–5 matches	reference		
6–10 matches	1.099	0.995–1.214	0.063
11–15 matches	1.081	0.930–1.257	0.309
16–20 matches	1.034	0.855–1.252	0.728
21–25 matches	0.842	0.648–1.095	0.200
26–30 matches	0.778	0.558–1.086	0.141
>30 matches	0.826	0.660–1.035	0.097

**bold**—highlights significant odds; a *p*-value of <0.05 was considered statistically significant.

## Data Availability

The data presented in this study are available on request from the corresponding author. The data are not publicly available due to privacy and ethical restrictions.
